# Flexibility in the Critical Period of Nutrient Sequestration in Bumble Bee Queens

**DOI:** 10.1093/iob/obab009

**Published:** 2021-04-19

**Authors:** Kristal M Watrous, Claudinéia P Costa, Yadira R Diaz, S Hollis Woodard

**Affiliations:** Department of Entomology, The University of California, Riverside, CA 92521, USA

## Abstract

**Synopsis:**

Bumble bee queens undergo a nutrient storage period prior to entering diapause wherein they sequester glycogen and lipids that are metabolized during overwintering. In the laboratory under optimal food availability conditions, the majority of nutrients are sequestered during the first few days of adulthood. However, if food resources are scarce during this narrow window of time, wild queen bumble bees might be limited in their ability to obtain adequate food resources for overwintering. Here we used a laboratory experiment to examine whether queen bumble bees exhibit flexibility in the timing of pre-overwintering nutrient sequestration, by limiting their access to either nectar (artificial) or pollen, the two primary foods for bumble bees, for varying periods of time. In response to these treatments, we quantified queen survival, changes in weight, and glycogen and lipids levels. We found evidence that queens are able to recuperate almost entirely from food resource limitation, with respect to nutrient storage, especially when it is experienced for shorter durations (up to 6 days). This study sheds light on how bumble bee queens are impacted by food resource availability at a critical life stage.

**Portuguese:**

As abelhas rainhas do gênero Bombus armazenam nutrientes antes de entrarem em diapausa, sequestrando o glicogênio e os lipídios que serão metabolizados durante o inverno. Em condições ideais de disponibilidade de alimento no laboratório, a maioria dos nutrientes é sequestrada nos primeiros dias de vida adulta. No entanto, em condições de escassez de alimento na natureza, as rainhas podem sofrer limitações em sua capacidade de obter recursos para o inverno. Nesse contexto, em condições controladas, examinamos se as rainhas exibem variações no sequestro de nutrientes, limitando o acesso ao néctar (artificial) ou pólen, seus principais alimentos, em diferentes intervalos de tempo. Em resposta a esses tratamentos, quantificamos a taxa de sobrevivência das rainhas, as mudanças no peso e os níveis de glicogênio e lipídios. Encontramos evidências de que as rainhas são capazes de recuperar a capacidade de armazenar nutrientes quase inteiramente, especialmente em períodos mais curtos de escassez de alimento (até 6 dias). Este estudo lança luz sobre como as rainhas são afetadas pela variação na disponibilidade de recursos alimentares em um estágio crítico da vida.

**Spanish:**

Las abejas reinas de generó Bombus, mejor conocidas como reinas de abejorro se someten a un período de almacenamiento de nutrientes antes de entrar en diapausa, en el cual secuestran glucógeno y lípidos que se metabolizan durante el invierno. En el laboratorio, en condiciones óptimas de disponibilidad de alimentos, la mayoría de los nutrientes se secuestran durante los primeros días de la edad adulta. Sin embargo, si los recursos alimenticios son escasos durante esta estrecha ventana de tiempo, las abejas reinas silvestres podrían verse limitadas en su capacidad para obtener recursos alimenticios adecuados para pasar el invierno. Aquí utilizamos un experimento de laboratorio para examinar si las abejas reinas exhiben flexibilidad en el momento del secuestro de nutrientes antes de la hibernación, al limitar su acceso al néctar (artificial) o al polen, los dos alimentos principales de los abejorros, durante períodos variables. En respuesta a estos tratamientos, cuantificamos la supervivencia de la reina, los cambios de peso y los niveles de glucógeno y lípidos. Encontramos evidencia de que las reinas pueden recuperarse casi por completo de la limitación de los recursos alimenticios, con respecto al almacenamiento de nutrientes, especialmente cuando se experimenta por períodos más cortos (hasta 6 días). Este estudio arroja luz sobre cómo las abejas reinas se ven afectadas por la disponibilidad de recursos alimenticios en una etapa crítica de la vida.

## Introduction

The bumble bees are a bee genus (*Bombus*) within the family Apidae that are important pollinators in many natural and agricultural ecosystems (Heinrich 2004; Goulson 2010). During the majority of the active nesting season, queen bumble bees remain within the nest, whereas workers forage to collect food resources and also perform work-related tasks in the nest. If nests are successful, they will grow in size and ultimately produce new reproductives (queens and males) at their last stages. Unlike the perennially social bees, such as the honey bees (genus *Apis*, family Apidae), most bumble bees are annually social and new nests are initiated each year. These new nests are founded by queen bumble bees who eclosed at the end of the previous season (typically in late summer or fall), mated, then overwintered in a diapause state. In spring, these queens emerge from overwintering to seek out nesting sites, thus beginning the new nesting season. Identifying life stage-specific stressors for bumble bees is a key goal for understanding the biology of this group and identifying the causal drivers of their widespread decline (Goulson et al. 2008; Woodard 2017).

Diapause is characterized as an arrested state of growth or development, and is a pre-programmed physiological and behavioral state that can be a particularly beneficial strategy for winter survival in insects (Danks 1991). During diapause, insects typically do not feed, and instead metabolize nutrients that were sequestered prior to the diapause period (Hahn and Denlinger 2007, 2011). Adult queens are the only bumble bee life stage and caste that undergo diapause; workers and males live only during the season in which they eclose, whereas queens typically have a longer life cycle of around one year. In preparation for overwintering in a diapause state, queens sequester copious amount of lipids and glycogen, primarily in trophocytes of the fat body (Alford 1969a; Röseler and Röseler 1986; Fliszkiewicz and Wilkaniec 2007; Votavová et al. 2015; Treanore and Amsalem 2020; but see Amsalem et al. 2015). Protein levels remain consistent and do not increase in preparation for diapause (Alford 1969b; Woodard et al. 2019; but see Treanore and Amsalem 2020). The fat body is a liver-like tissue in insects that is the primary location of energy storage and release and also plays important roles in detoxification and immune function (Arrese and Soulages 2010; Hahn and Denlinger 2011). The lipids and glycogen that bumble bee queens sequester during the pre-overwintering period are largely metabolized during diapause (Alford 1969a, 1969b; Holm 1972; Prîdal and Hofbauer 1996) and are critical for diapause survival (Beekman et al. 1998, 2000; Bogo et al. 2017; Woodard et al. 2019). Access to both pollen (the primary source of dietary fatty acids) and nectar (the primary source of sugars stored as glycogen) are critical for overwintering survival. Queen mass upon entering diapause has been positively associated with diapause survival in a number of studies (Beekman et al. 1998, 2000; Bogo et al. 2017; Treanore and Amsalem 2020), and a lack of either nectar-derived sugar or pollen in the diet during the pre-diapause period significantly reduces overwintering survival in laboratory-reared queens (Woodard et al. 2019). There are also sublethal consequences of pre-diapause food availability. Sugar-poor diets during the pre-overwintering period cause a decrease in the expression of detoxification enzymes in the fat body, which suggests some impairment in this function under scenarios where floral nectar is limited (Costa et al. 2020).

Stereotypical physiological events are often programmed parts of development, but they can also be responsive to ecological conditions or unpredictable events (Wingfield et al. 1998; Flatt and Heyland 2011). This flexibility allows organisms to coordinate their behavior and physiology with their surroundings, respond dynamically to environmental change, and ultimately persist. Prior to leaving their natal nests, queens feed exclusively on food stores in the nest, which have been collected by their sister-workers from the area around the nest site. Typically this ranges from a radius of a few meters up to 10 km (Chapman et al. 2003; Darvill et al. 2004; Knight et al. 2005; Charman et al. 2010; Rao and Strange 2012; Jha and Kremen 2013; Geib et al. 2015). The majority of glycogen and lipids are sequestered during the first ∼3 days of adulthood, then levels plateau before or around day 6 (Alford 1969a; Woodard et al. 2019; Treanore and Amsalem 2020). These timepoints correspond with the ages that queens are primarily still in their natal nests, and correspond with the timepoint around which pollen consumption by queens (of *B. terrestris*) declines dramatically (Přidal and Hofbauer 1996). Around the adult age of 3–5 days, queens become competent to fly, which is a prerequisite for leaving their natal nests (Sladen 1912; Goulson 2010). Upon leaving the nest, queens can access food resources at spatial scales beyond the 10 km surrounding their natal nests (Lepais et al. 2010), before entering their overwintering hibernacula. This potential increase in the area from which food resources are available to queens might allow them to compensate for poor late-season food resources in the areas around their natal nests by seeking out more foraging opportunities at greater distances. Such compensation might be especially important when there are late-season declines in floral resources (Timberlake et al. 2019), which may be contributing to the loss of pollinator species that are active in late summer (Balfour et al. 2018).

Here, we explored flexibility in the critical nutrient storage period preceding overwintering in queens of the common eastern bumble bee, *Bombus impatiens*. Specifically, we tested the ability of adult bumble bee queens to recover from diets that are temporarily deficient in either pollen or artificial nectar (for 3, 6, or 9 days) during the early-life, pre-diapause nutrient sequestration period. To examine temporary deficiency in food availability, we starved queens for one these durations, then changed their diet so that they were provided with food *ad libitum*. In response to these diet treatments, we monitored survival and changes in queen weight across the test period, and also quantified abdominal nutrient levels (glycogen and lipids) at the end of the test period. Based on the hypothesis that flexibility in the timing of nutrient sequestration is advantageous for bumble bee queens, we predicted that queens that experienced short periods of nutritional dearth would recuperate their nutrient stores, if they were subsequently switched to diets containing abundant pollen and artificial nectar. However, we also predicted that we might see limits to this flexibility when queens are subjected to longer periods of nutritional dearth.

## Materials and methods

### Bee rearing and diet treatment administration

All queens used in the experiment (*n *=* *104) were obtained from natal source colonies (*n *=* *8) provided by Koppert Biological Systems (Romulus, MI). Colonies were housed in an insectary room in the UC Riverside Entomology Building maintained at room temperature (∼21°C), uncontrolled humidity conditions (but >40% RH), and under constant darkness or red light, which is not visible to bees. All colonies were provided with mixed-source, honey bee-collected pollen supplied by Biobest USA, Inc. that was mixed with artificial nectar syrup supplied by Koppert Biological Systems. Both pollen and artificial nectar were provided to whole colonies *ad libitum*.

We removed newly-eclosed queens from their natal colonies on the day of eclosion (identified based on their silvery appearance), weighed them to determine their initial adult weight, and then contained them in plastic rearing cages (approximately W15 × D15 × H10 cm). On the day that they were removed from their natal colonies and for the duration of the experiment, the queens were maintained in a rearing room in the UC Riverside Insectary and Quarantine Facility maintained at 28°C and 80% RH and under red light. Queens were unable to fly or forage during the experiment.

At the time the queens were removed from their natal colonies, they were assigned to one of nine diet treatments wherein they were either provided with pollen (as described above) and artificial nectar (a 50% w/v sucrose solution) for the entire duration of the experimental period (the control diet), or they were deprived of either artificial nectar (nectar-starved diets, hereafter “NS”) or of pollen (pollen-starved diets, hereafter “PS”) for some duration of time (either 3, 6, 9, or 12 days; Supplementary Table S1). Following their assigned period of pollen or nectar starvation (with the exception of the 12-day group) the queens were changed to the control diet. We subsequently refer to this change in diet as a diet switch, which refers to the experimental change of queens from one of these types of temporary starvation (either pollen or nectar, for one of the three durations) to the control diet. Our NS3d queens, for example, refer to their being nectar starved for 3 days; these queens received pollen for the duration of the experiment but were starved of artificial nectar for 3 days, then were switched to the control nectar diet for the remainder of the experiment. *Bombusimpatiens* queen nutrient stores completely plateau by around age 12 days (Alford 1969a; Röseler and Röseler 1986; Woodard et al. 2019; Treanore and Amsalem 2020), which might signify preparation for or the onset of diapause (Costa et al. 2020). We chose the timepoints of 3, 6, 9, and 12 days of age because they span the approximately 12-day pre-diapause, nutrient storage period. Queens that were starved of artificial nectar received pollen (the same source as described above) mixed with water and also water (0% sucrose solution) in the liquid feeder, in lieu of artificial nectar. These queens received a trivial amount of sugar in their diet because honey bee-collected pollen contains some nectar that is added when pollen is collected into corbiculae (Roulston and Cane 2000). However, this small amount of sugar is insufficient for queens to sequester glycogen in the amounts observed in queens fed a 50% (w/v) sucrose solution during the pre-overwintering period (Woodard et al. 2019). Queens in the pollen-starved group received 50% (w/v) sucrose solution and did not receive pollen. This diet was entirely deficient in lipids and protein. Our diet treatment groups were designed to reflect realistic nutritional scenarios for bumble bee queens. In wild flowering plants, nectar sugar concentrations typically range from 25% to 75%, with values often close to 40% (Chalcoff et al. 2006; Pamminger et al. 2019), whereas nectar stored within nests contains sugar at higher concentrations (Knee and Medler, 1965). Bees can experience pollen and nectar scarcity; landscapes can be limited in the availability of these floral rewards (Timberlake et al. 2019), and bumble bees can consume the entirety of their stored pollen and nectar within a few days if they are unable to forage and replenish stored food in the nest (Cartar and Dill 1990; Heinrich 2004; Couvillon and Dornhaus 2010).

Where applicable, every 2 days, we gave the queens fresh pollen and the liquid feeder (containing either sucrose solution or water) was replaced to prevent spoilage. During the 12-day treatment administration period, any queens in the nectar-starved and pollen-starved groups that underwent a diet switch were changed to the control diet between 800 and 1000 h on the day of the switch. Queens were not mated in the experiment in order to avoid introducing effects of mating; queens sequester nutrients during early adulthood regardless of mating status (Alford 1969a), and both mated and virgin queens are able to survive diapause (Greeff and Schmid-Hempel 2008; Bogo et al. 2017).

### Survival, weight gain, and body size

All queens were inspected daily for mortality. We examined weight gain as a proxy for nutrient storage. All queens were weighed initially; at ages 3, 6, and 9 days; and at the end of the experiment (at age 12 days) to examine weight changes during the 12-day nutrient sequestration period. For any queens that died during the experiment, we excluded weight data from the day these queens died and also from the day prior to death, to exclude weight changes that might be associated with metabolic dysregulation or the onset of death. We also excluded data from individuals that were nectar-starved for more than 6 days from this analysis because few or no bees from the 9 and 12 days treatment groups, respectively, survived to the end of the experiment. Changes in mass are not solely reflective of changes in nutrient levels because they are confounded by the presence of food in the gut and changes in water metabolism; however, they do provide an indication of whether or not nutrient storage might be occurring (Woodard et al. 2019). All queens in the experiment that lived to the age of 12 days (*n *=* *75) were weighed a final time then collected into a −80°C freezer until they were used for nutritional analyses. In our statistical analyses, we examined incremental weight change between 3-day periods and also the total change in queen weight across the entire nutrient sequestration period. We also used *posthoc* tests to compare weight data within days, between treatment groups. We estimated adult (final) body size for all queens in the experiment and included these data as a covariate in our statistical analyses. For this, forewings were removed and the length of the marginal cell of each wing was measured, then the measurements were averaged for each queen as a proxy for overall body size (Knee and Medler 1965; Owen 1988; Yerushalmi et al. 2006; Shpigler et al. 2013). Hereafter, “body size” refers to the average length of the two marginal cells from an individual queen.

### Nutrient quantitation

We quantified lipid and glycogen levels for a subset of queens that lived for the duration of the 12-day nutrient sequestration period (*n *=* *61 and 52, respectively). Any queens that died during the experiment (*n *=* *29) were not included in the nutrient quantitation analysis. We also excluded individuals that were nectar-starved for more than 6 days from this analysis because so few bees from these treatment groups survived to the end of the experiment. We did not quantify protein levels, given that these do not change in bumble bee queens prior to overwintering (Alford 1969b; Woodard et al. 2019), nor are protein levels impacted by pollen and nectar starvation (Woodard et al. 2019).

First, we separated abdomens from the rest of the body and removed digestive tracts. This allowed us to quantify lipid and glycogen levels primarily in the fat body, where the majority of nutrients are stored, and also the ovaries, which are undeveloped at this life stage (Geva et al. 2005; Amsalem et al. 2015). Hereafter, we refer to this tissue as the abdomen. Dissected abdomens were dried for 5 days in a drying oven at 50°C until dry weight stabilized. This weight was recorded as the final abdominal dry mass. Next, queen abdomens were homogenized in a 2 mL vial with 1.5 mL water and two chrome beads, shaking for 5 min at 30 shakes/s in a Qiagen TissueLyser II. We quantified abdominal lipid concentrations within the organic fraction using a sulfo-phospho-vanillin assay adapted from Cheng et al. (2011) and McMahon et al. (2013) and used previously in Woodard et al. (2019). Here, we used cholesterol standards (Sigma–Aldrich; 175, 150, 125, 100, 75, 50, and 25 μg/mL), which are commonly used for lipid quantification in insects, but can slightly underestimate total lipid amounts (Williams et al. 2011). Glycogen was first separated from total sugars in the aqueous fraction of homogenized tissue concentrations through sodium sulfate precipitation, then glycogen was quantified using an anthrone-based assay adapted from Leyva et al. (2008) using glucose standards (Rica; 210, 180, 150, 120, 90, 60, and 30 μg/mL). Methods for these assays are provided in greater detail in Woodard et al. (2019). Quantitation of samples was performed on a Varioskan LUX microplate reader. Samples were assayed in triplicate, then coefficients of variation (CV) were calculated for each triplicate; mean CV values averaged 8.10% and 15.88% for lipids and glycogen concentrations, respectively. Following this, we also removed outliers based on a 95% confidence interval, then multiplied the mean sample values by the dilution factor to estimate macronutrient concentrations (mg/mL) for each sample.

### Statistical analyses

All statistical analyses were performed in R version 3.6.1 (R Core Team 2019) and only *P*-values < 0.05 were considered significant. All results were visualized with the “ggplot2” package (Wickham 2011). After identifying normality or non-normality of the data using the Shapiro–Wilk test, we used either parametric and non-parametric tests in our statistical analyses, as outlined below. For survival, the likelihood of queen mortality was compared across the diet treatments using Cox Proportional Hazards model (function coxph in package “survival”; Therneau 2015; Therneau and Grambsch 2000) with a robust standard error call. Total weight change data were also compared between diet treatment groups using linear mixed effects models (LMMs). Nutrient concentrations were analyzed using generalized linear mixed models (GLMMs) for lipids, and LMMs for glycogen. These were performed with the glmer and lmer function in the R package lme4 version 1.1-10 (Bates et al. 2015). For all analyses, queen diet treatment, starting mass, abdominal dry mass, and body size were all evaluated as potential fixed factors to be included in our statistical models, if they were components of best-fit models. The best-fit models for our data were selected based on the Akaike’s Information Criterion (AIC) using the “dredge” command within the MUMIn package (Barton 2015). Main effects were computed using an ANOVA with a *χ*^2^-test. Following model selection, factors of interest were also analyzed by performing likelihood ratio tests (LRTs) comparing the models with factors to a null model without these factors. *Posthoc t*-tests were conducted using Tukey’s multiple comparison of means. Outliers (data points outside of 95% confidence interval) were removed from the nutritional data before analysis.

## Results

### Survival across experiment

**Fig. 1:**
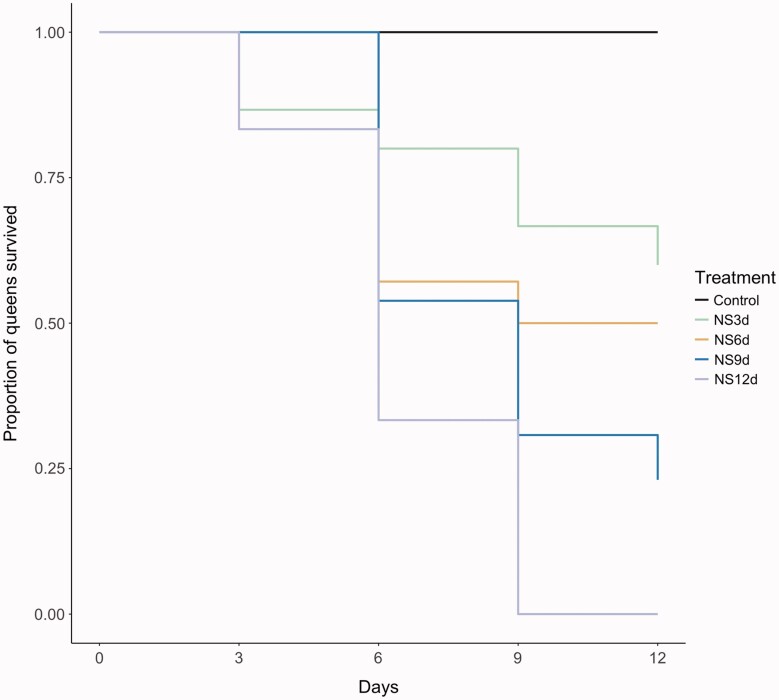
Queen survival as a function of diet treatment. Nectar starvation significantly impacted queen mortality (Pr(>|*z*|) < 0.0001) relative to the control treatment. Queens were deprived of artificial nectar for either 3 (NS3d), 6 (NS6d), 9 (NS9d), or 12 (NS12d) days. The proportion of queens surviving at each timepoint is shown for the control group and nectar-starved groups only, as all bees in the pollen-starved groups survived the entirety of the experiment (Pr(>|*z*|) > 0.05) for control and all pollen-starved treatments.

Nectar starvation had a significant impact on queen mortality during the 12-day nutrient sequestration period, as queens starved of sugar for any duration (3, 6, 9, or 12 days) exhibited a substantial increase in mortality relative to queens provided the control diet (Pr (>|*z*|) values for all comparisons < 0.0001; Supplementary Table S2 and Fig. 1). Pollen starvation did not impact survival; all pollen-starved bees survived for the full duration of the experiment (all Pr (>|*z*|) values > 0.05; Supplementary Table S2). The best-fit model to analyze queen survival included diet treatment, and the interaction between starting mass and body size as fixed factors; this interaction between body size and starting mass also significantly impacted queen mortality (Pr (>|*z*|) < 0.05; Supplementary Table S2).

### Weight gain

**Fig. 2:**
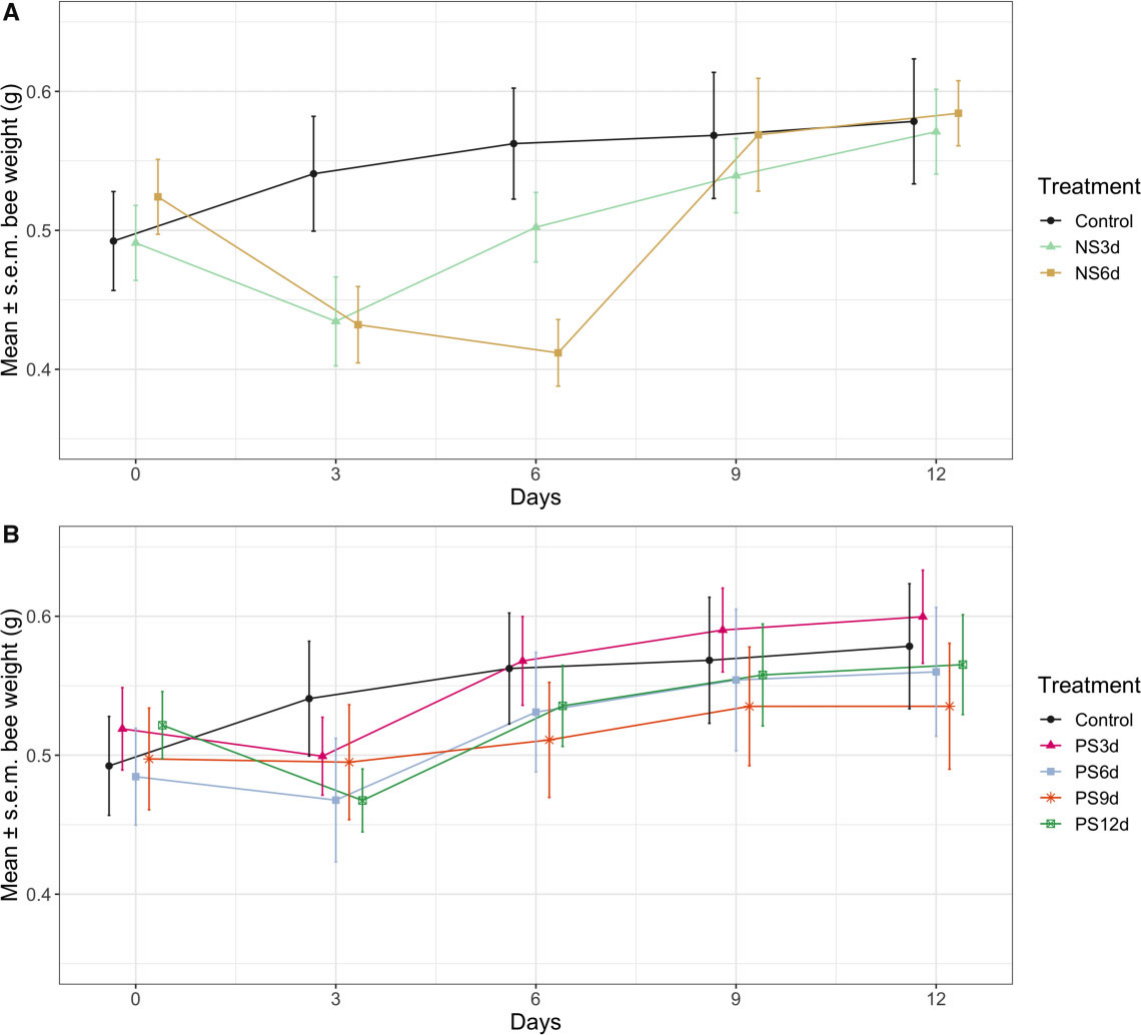
Weight change through time as a function of diet treatment. (**A**) Nectar starvation treatment groups, (**B**) Pollen starvation groups. Bee weight was recorded every 3 days and averaged within each treatment for each timepoint. Data for the control diet group is shown (in black) for both A and B. Sample sizes (with the percent that survived until the end of the 12-day experiment in parentheses) are as follows: control: 10 (100%); NS3d: 15 (60%); NS6d: 14 (100%); NS9d: 13 (23%); NS12d: 6 (0%); PS3d: 9 (100%); PS6d: 13 (100%); PS9d: 13 (100%); and PS12d: 11 (100%). Across days, the overall effect of diet (all treatment groups) on weight change was significant (Table 1). Within days, the NS6d group was significantly lower than the control group on day 6 (see Supplementary Fig. S1).

We conducted separate analyses for our nectar-starved and pollen-starved groups in our examination of weight gain. For nectar-starved groups, we employed a best-fit model that included the interaction between diet treatment and day as fixed factor, and individual (sample ID) and natal colony as random factors (LMM: comparison of the model with null model: LRT: *χ*^2^ = 111.72, d.f. = 14, *P* < 0.001; Table 1). With respect to our nectar starvation treatment groups, there was significant, consistent weight gain across the 12 day period (Tukey’s *posthoc*: Pr (>|*z*|) values for all comparisons to day 0 < 0.01; Fig. 2A, Table 1, and Supplementary Table S3). This analysis included the control, NS3d, and NS6d groups (NS9d and NS12d groups were excluded due to low sample sizes). In our comparison of weight data within days, between treatment groups, there was a significant difference between the NS6d and control diet group on day 6, where the NS6d queens had lower weights (*P*-value for this comparison 0.0088; Supplementary Fig. S1). For pollen-starved groups, the best-fit model included the interaction between diet treatment and day as fixed factors, and individual and natal colony as random factors (LMM: comparison of the model with null model: LRT: *χ*^2^ = 207.55, d.f. = 24, *P* < 0.001, Table 1). Here too, there was significant, consistent weight gain across the 12 day period (Tukey’s *posthoc*: Pr (>|*z*|) values for all comparisons to day 0 < 0.01; Fig. 2B, Table 1, and Supplementary Table S3). Within days, there were no significant differences between our pollen starvation treatment groups and the control diet group (Supplementary Fig. S1).

**Table 1:** The main effects for weight gain in queens that received a nectar-starved or pollen-starved diet from an ANOVA

	*χ* ^2^	D.f.	Pr (>*χ*^2^)
Nectar			
Treatment	4.633	2	0.099
Timepoint	101.763	4	<0.001***
Treatment: timepoint^+^	89.064	8	<0.001***
Pollen			
Treatment	9.942	4	0.041*
Timepoint	266.293	4	<0.001***
Treatment: timepoint^+^	62.42	16	<0.001***

Queens were deprived of either artificial nectar or pollen for some duration of time (either 3, 6, 9, or 12 days). Asterisks indicate statistical significance; significance codes: “***” 0.001, “**” 0.01, “*” 0.05. “^*+*^” the interaction term between type of diet (Treatment) and ages that queens were weighed (timepoint).

### Nutrient levels

**Fig. 3:**
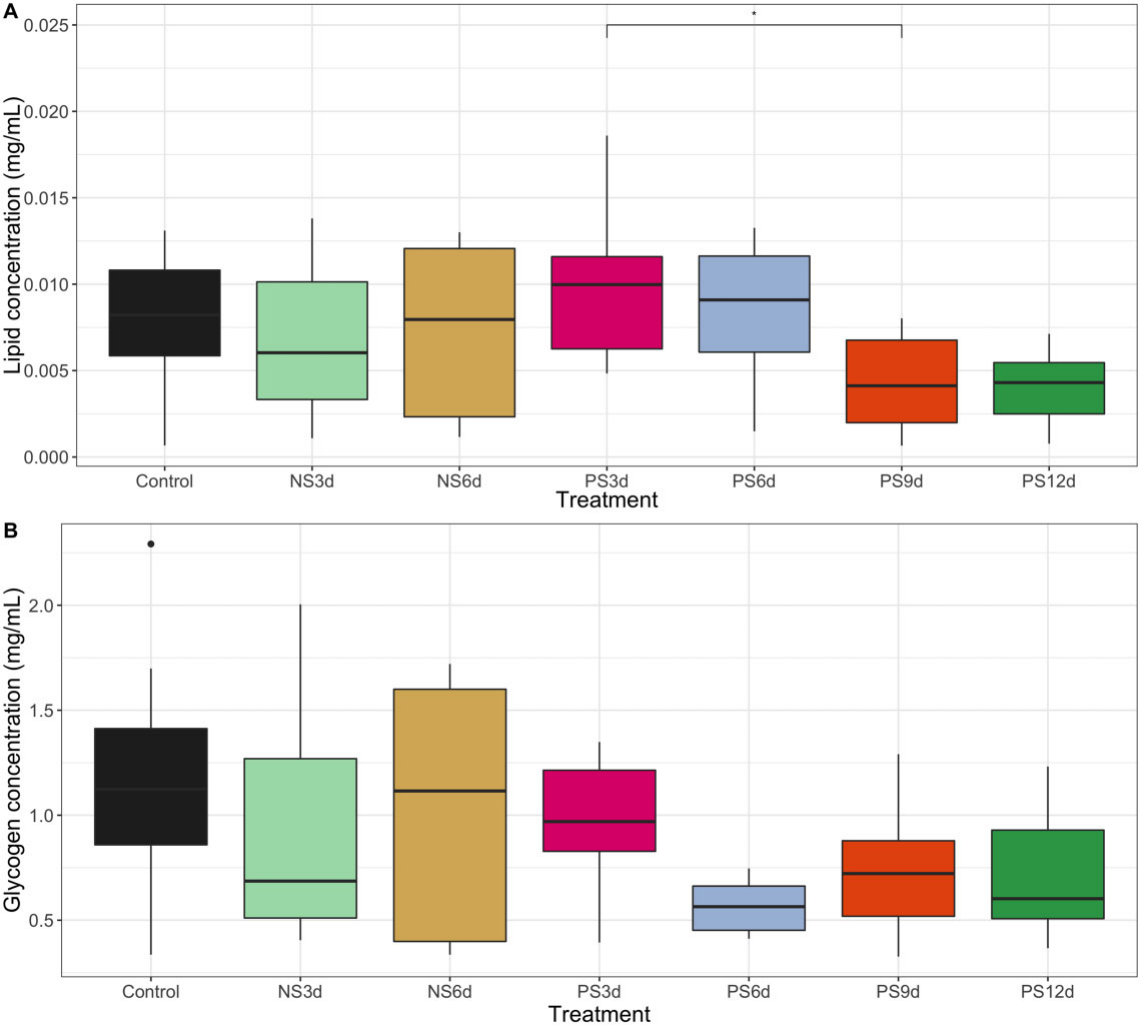
Total abdominal nutrient levels at the end of treatment administration period. (**A**) Lipid levels for all treatment groups, normalized by dried bee abdomen mass. (**B**) Glycogen levels for all treatment groups, normalized by dried bee abdomen mass. Data were log-transformed for analysis to meet assumptions of normality. Data not shown for bees starved of nectar for more than 6 days because so few bees from these treatment groups survived to the end of the experiment. Queens were deprived of either artificial nectar (nectar-starved diets, hereafter “NS”) or of pollen (pollen-starved diets, hereafter “PS”) for some duration of time (either 3, 6, 9, or 12 days).

Lipid concentrations were analyzed with a best-fit model that included treatment as a fixed factor and colony as a random factor. Diet treatment had a significant impact on queen abdominal lipid levels across the 12-day period (GLMM: comparison of the model with null model: LRT: *χ*^2^ = 16.495, d.f. = 6, *P* = 0.011; Fig. 3A). Queens that were starved of pollen for 9 days had significantly lower lipid levels than queens starved of pollen for only 3 days (Tukey’s *posthoc*: PS9d versus PS3d: *z*-value = 3.040, Pr (>|*z*|) = 0.035; Supplementary Table S5), but were not significantly different from the control group (Tukey’s *posthoc*: PS9d versus control diet: *z*-value = 2.285, Pr (>|*z*|) = 0.236; Supplementary Table S5). Queens that were starved of pollen for shorter durations (3 or 6 days) or for a longer duration of 12 days, or starved of artificial nectar for any duration, did not have significantly different lipid concentrations from queens in the control group (Supplementary Table S5). For glycogen concentration, employing a best-fit model that included the abdominal dry mass as a fixed factor and colony as a random factor, we found that abdominal glycogen levels were not significantly impacted by diet treatment (Fig. 3B and Supplementary Table S6), but were significantly associated with the abdominal dry mass (LMM: comparison of the model with null model: LRT: *χ*^2^ = 10.883, d.f. = 1, *P* < 0.001). This analysis only included queens in the control, NS3d, and NS6d groups, given the high mortality observed in the NS9d and NS12d groups.

## Discussion

Identifying the quantity and diversity of nutrients that queens need to complete their life cycle successfully is a critical topic in bumble bee conservation physiology (Woodard 2017). Diapause survival is impacted by pollen and nectar availability in the period preceding diapause (Woodard et al. 2019), and is positively associated with queen weight at the time they enter diapause (Beekman et al. 1998, 2000, Bogo et al. 2017; Treanore and Amsalem 2020). Our analyses revealed that bumble bee queens have the capacity to recuperate from periods of nutritional dearth during the period preceding diapause, with respect to nutrient storage. Thus, queens exhibit some flexibility in the timing of nutrient sequestration, which has previously been reported to occur by age ∼3–6 days in multiple species (Alford 1969a; Röseler and Röseler 1986), including *B. impatiens* (Woodard et al. 2019; Treanore and Amsalem 2020). Queens in our study that were deprived of artificial nectar for the first 3 days of adulthood lost weight during this period, whereas queens on the control diet gained weight during this time. However, upon switching to the control diet at the age of 3 days, these queens began to gain weight and they continued to do so until the end of the experiment, with a final mean weight that was not different from the control bees. Queens that were starved of nectar for a longer duration of 6 days exhibited a similar pattern in that when they were switched to a control diet on day 6, by day 9, their weight resembled that of control queens and ultimately did not differ from the control group. Queens in both of these nectar-starved groups also had glycogen levels that did not differ from the control group. From our study, it is unclear whether queens deprived of nectar-derived sugar for even longer periods (9 or 12 days) are also potentially able to recuperate their nutrient stores after this form of nutritional stress, given our limited sample sizes due to high mortality in these treatment groups. Queens deprived of pollen for longer durations (9 or 12 days) gained less weight overall than the control diet queens, but their lipid levels were not lower than the control set of queens. This suggests that queens are able to recover from both pollen and nectar limitation experienced during the pre-diapause period, at least for the durations that we explored in our experiment.

As we only examined weight and nutrient storage in our experiment, there might be additional sublethal consequences of starvation that we did not examine, which impact queen fitness. For example, limitation of nectar-derived sugar in the bumble bee queen diet during the pre-diapause period results in a downregulation of P450 enzyme expression, suggesting an impairment of immune function (Costa et al. 2020). Similar relationships between diet and immunity have been detected in other studies of bumble bees (Roger et al. 2017) and honey bees (Alaux et al. 2010; Corby-Harris et al. 2019). Additionally, the susceptibility of bumble bee queens to pesticides, such as neonicotinoids, might then be greater when they feed on particular pollen diets (Leza et al. 2018). Further exploration of the sublethal effects of temporary starvation is warranted to better understand the breadth of impacts of nutritional stress on bumble bee queen health.

Following periods of starvation, the queens in our study may have increased their consumption in order to sequester sufficient nutrients for overwintering. Compensatory feeding following periods of inadequate nutrition has been broadly demonstrated in vertebrate (Lochmiller et al. 2000; Johansen et al. 2001) and invertebrate (Rueda et al. 1991; Wheeler and Slansky 1991; Lavoie and Oberhauser 2004; Berner et al. 2005) taxa. Although the phenomenon has not been explored in individual bumble bees, there is evidence that workers regulate their intake of particular nutrients (Stabler et al. 2015; Vaudo et al. 2016). Thus, queens may also have the capacity to regulate their feeding (either food quantity or composition) as a means of achieving nutritional homeostasis. The ability to upregulate feeding might be particularly important during the period preceding diapause, given that failure to sequester adequate nutrients decreases the likelihood of surviving diapause (Woodard et al. 2019). There is also some evidence that queen nutritional status is positively associated with the onset of diapause, suggesting that nutrient sensing might contribute to preparation for, or entry into, diapause (Costa et al. 2020). Queens may also synthesize fatty acids from dietary sugar, and may convert fatty acids to glycogen via gluconeogenesis; these biochemical processes may allow queens to convert nutrients from nectar and pollen, respectively, to compensate for a lack of pollen or nectar in the diet. This might be an alternative or complementary mechanism through which queens can mediate the effects of short-term food limitation, and might have contributed to our finding that queens in our experiment did not exhibit reduced lipid levels even when starved of pollen for the entire 12-day duration.

Broadly, our findings suggest that bumble bee queens can endure periods of nutritional dearth during early adulthood, via delayed nutrient sequestration before entering diapause. This extended window of time in which to store nutrients may allow queens to access food resources from broader spatial scales once they leave their natal nests. Thus, our study sheds light on one way that bumble bee queens might overcome food unavailability during early adulthood. However, in the wild, recovery would be dependent on the ability to find and access food resources, and might also be negatively impacted by the amount of energy use required to acquire those resources. Further, sublethal effects of inadequate nutrition are only beginning to be understood for bumble bees (Moerman et al. 2016; Dance et al. 2017; Roger et al. 2017; Leza et al. 2018; Woodard et al. 2019; Costa et al. 2020), and might translate to reduced fitness for queens, particularly in landscapes where they are exposed to multiple stressors (Dance et al. 2017; Leza et al. 2018; Costa et al. 2020). Identifying the full breadth of ways that queen bumble bees respond to nutritional challenges, across all life stages, will help us better understand patterns of persistence and decline in this important pollinator group.

## Funding

This working was supported by funding from USDA NIFA (CA-R-ENT-5122-H to S.H.W.).

## Data availability statements

All analyses and pipelines for this study can be found on GitHub (https://github.com/claudinpcosta/2021-Queen-nutrient-storage-timing).

## Supplementary data

Supplementary data are available at *IOB* online.

## Conflict of interest statement

None declared.

## Supplementary Material

obab009_Supplementary_Data
