# Supplementary material for: Flexibility in the Critical Period of Nutrient Sequestration in Bumble Bee Queens
Source: Integr Org Biol. 2021 Apr 19;3(1):obab009. doi: 10.1093/iob/obab009 (PMC8179628; doi:10.1093/iob/obab009)
Supplement: obab009_Supplementary_Data [file obab009_supplementary_data.zip › Portuguese abstract.docx]

**Portuguese**

As abelhas rainhas do gênero Bombus armazenam nutrientes antes de entrarem em diapausa, sequestrando o glicogênio e os lipídios que serão metabolizados durante o inverno. Em condições ideais de disponibilidade de alimento no laboratório, a maioria dos nutrientes é sequestrada nos primeiros dias de vida adulta. No entanto, em condições de escassez de alimento na natureza, as rainhas podem sofrer limitações em sua capacidade de obter recursos para o inverno. Nesse contexto, em condições controladas, examinamos se as rainhas exibem variações no sequestro de nutrientes, limitando o acesso ao néctar (artificial) ou pólen, seus principais alimentos, em diferentes intervalos de tempo. Em resposta a esses tratamentos, quantificamos a taxa de sobrevivência das rainhas, as mudanças no peso e os níveis de glicogênio e lipídios. Encontramos evidências de que as rainhas são capazes de recuperar a capacidade de armazenar nutrientes quase inteiramente, especialmente em períodos mais curtos de escassez de alimento (até 6 dias). Este estudo lança luz sobre como as rainhas são afetadas pela variação na disponibilidade de recursos alimentares em um estágio crítico da vida.
