# Supplementary material for: Flexibility in the Critical Period of Nutrient Sequestration in Bumble Bee Queens
Source: Integr Org Biol. 2021 Apr 19;3(1):obab009. doi: 10.1093/iob/obab009 (PMC8179628; doi:10.1093/iob/obab009)
Supplement: obab009_Supplementary_Data [file obab009_supplementary_data.zip › Spanish abstract.docx]

**Spanish**

Las abejas reinas de generó Bombus, mejor conocidas como reinas de abejorro se someten a un período de almacenamiento de nutrientes antes de entrar en diapausa, en el cual secuestran glucógeno y lípidos que se metabolizan durante el invierno. En el laboratorio, en condiciones óptimas de disponibilidad de alimentos, la mayoría de los nutrientes se secuestran durante los primeros días de la edad adulta. Sin embargo, si los recursos alimenticios son escasos durante esta estrecha ventana de tiempo, las abejas reinas silvestres podrían verse limitadas en su capacidad para obtener recursos alimenticios adecuados para pasar el invierno. Aquí utilizamos un experimento de laboratorio para examinar si las abejas reinas exhiben flexibilidad en el momento del secuestro de nutrientes antes de la hibernación, al limitar su acceso al néctar (artificial) o al polen, los dos alimentos principales de los abejorros, durante períodos variables. En respuesta a estos tratamientos, cuantificamos la supervivencia de la reina, los cambios de peso y los niveles de glucógeno y lípidos. Encontramos evidencia de que las reinas pueden recuperarse casi por completo de la limitación de los recursos alimenticios, con respecto al almacenamiento de nutrientes, especialmente cuando se experimenta por períodos más cortos (hasta 6 días). Este estudio arroja luz sobre cómo las abejas reinas se ven afectadas por la disponibilidad de recursos alimenticios en una etapa crítica de la vida.
