# Supplementary material for: Flexibility in the Critical Period of Nutrient Sequestration in Bumble Bee Queens
Source: Integr Org Biol. 2021 Apr 19;3(1):obab009. doi: 10.1093/iob/obab009 (PMC8179628; doi:10.1093/iob/obab009)
Supplement: obab009_Supplementary_Data [file obab009_supplementary_data.zip › Supplementary material.docx]

**Supplementary material for Integrative Organismal Biology**

Title: Flexibility in the critical period of nutrient sequestration in bumble bee queens

Authors: Kristal M. Watrous, Claudinéia Costa, Yadira R. Diaz, S. Hollis Woodard

**Table S1.** **Sample counts by colony and treatment**. Queens were deprived of either artificial nectar (nectar-starved diets, hereafter “NS”) or pollen (pollen-starved diets, hereafter “PS”) for some duration of time (either three, six, nine, or twelve days).

|  | |  | **Colonies** | | | | | | | |
| --- | --- | --- | --- | --- | --- | --- | --- | --- | --- | --- |
|  | |  | **BS01** | **BS05** | **DR2** | **MP10** | **QP2** | **QP3** | **WP8** | **WP9** |
| **Treatments** | **Control** | |  | 4 | 1 |  | 2 | 3 |  |  |
|  | **NS3d** | | 1 | 5 | 1 | 1 | 2 | 2 | 1 | 2 |
|  | **NS6d** | |  | 5 | 1 | 1 | 2 | 4 | 1 |  |
|  | **NS9d** | |  | 4 | 1 | 1 | 2 | 5 |  |  |
|  | **NS12d** | |  |  | 1 |  | 2 | 3 |  |  |
|  | **PS3d** | |  | 3 | 1 |  | 2 | 3 |  |  |
|  | **PS6d** | | 1 | 4 |  |  | 1 | 5 | 1 | 1 |
|  | **PS9d** | | 1 | 4 |  | 2 | 1 | 5 |  |  |
|  | **PS12d** | |  | 4 | 1 |  | 2 | 3 | 1 |  |

**Table S2. Cox Proportional Hazards output for survival analysis.** Hazard ratio indicates the likelihood of the hazard, in this case, the likelihood of a bee dying based on diet treatment. Hazard ratio (HR) of one is no effect; HR < 1 is a reduction in hazard (decreased likelihood of dying); HR > 1: an increase in hazard (increased likelihood of dying). Asterisks indicate statistical significance; significance codes: ‘***’ 0.001, ‘**’ 0.01, ‘*’ 0.05. Queens were deprived of either artificial nectar (nectar-starved diets, hereafter “NS”) or pollen (pollen-starved diets, hereafter “PS”) for some duration of time (either three, six, nine, or twelve days).

| **Term** | **Estimate (Hazard Ratio)** | **Robust Standard error** | **Statistic (z-value)** | **Pr(>\|z\|)** | **Confidence interval (low)** | **Confidence interval (high)** |
| --- | --- | --- | --- | --- | --- | --- |
| TreatmentNS3d | 9.67E+08 | 4.14E-01 | 49.949 | <2e-16 *** | 4.30E+08 | 2.18E+09 |
| TreatmentNS6d | 1.66E+09 | 4.15E-01 | 51.102 | <2e-16 *** | 7.33E+08 | 3.74E+09 |
| TreatmentNS9d | 2.64E+09 | 3.90E-01 | 55.582 | <2e-16 *** | 1.23E+09 | 5.68E+09 |
| TreatmentNS12d | 4.88E+09 | 3.90E-01 | 57.145 | <2e-16 *** | 2.27E+09 | 1.05E+10 |
| TreatmentPS3d | 9.48E-01 | 4.02E-01 | -0.134 | 0.894 | 4.32E-01 | 2.08E+00 |
| TreatmentPS6d | 1.53E+00 | 5.72E-01 | 0.747 | 0.455 | 5.00E-01 | 4.70E+00 |
| TreatmentPS9d | 1.28E+00 | 5.29E-01 | 0.462 | 0.644 | 4.53E-01 | 3.60E+00 |
| TreatmentPS12d | 1.10E+00 | 4.27E-01 | 0.213 | 0.831 | 4.75E-01 | 2.53E+00 |
| Body size | 4.86E-25 | 2.02E+01 | -2.767 | 0.005 ** | 2.93E-42 | 8.05E-08 |
| Starting Weight | 4.17E-03 | 2.15E+00 | -2.553 | 0.010 * | 6.20E-05 | 2.80E-01 |
| StartWt:WingAvgmm | 4.18E+05 | 4.86E+00 | 2.661 | 0.008 ** | 3.03E+01 | 5.77E+09 |

**Table S3.** **Pairwise comparisons from weight gain in queens that received a nectar-starved diet with Tukey contrasts**. Queens were deprived of artificial nectar (nectar-starved diets, hereafter “NS”) for some duration of time (either three, six, nine, or twelve days). Asterisks indicate statistical significance; significance codes: ‘***’ 0.001, ‘**’ 0.01, ‘*’ 0.05.

|  | **Tukey contrasts** | **Estimate** | **Standard Error** | **z-value** | **Pr(>\|z\|)** |
| --- | --- | --- | --- | --- | --- |
| **Treatment** | NS3d-control | 0.005 | 0.035 | 0.156 | 0.987 |
|  | NS6d-control | 0.002 | 0.041 | 0.072 | 0.997 |
|  | NS6d-NS3d | 0.002 | 0.042 | -0.060 | 0.998 |
| **Days** | 3-0 | 0.04841 | 0.01631 | 2.968 | 0.025 * |
|  | 6-0 | 0.07009 | 0.01631 | 4.297 | < 0.001 *** |
|  | 9-0 | 0.076 | 0.01631 | 4.66 | < 0.001 *** |
|  | 12-0 | 0.0861 | 0.01631 | 5.279 | < 0.001 *** |
|  | 6-3 | 0.02168 | 0.01631 | 1.329 | 0.673 |
|  | 9-3 | 0.02759 | 0.01631 | 1.692 | 0.439 |
|  | 12-3 | 0.03769 | 0.01631 | 2.311 | 0.141 |
|  | 9-6 | 0.00591 | 0.01631 | 0.362 | 0.996 |
|  | 12-6 | 0.01601 | 0.01631 | 0.982 | 0.864 |
|  | 12-9 | 0.0101 | 0.01631 | 0.619 | 0.972 |

**Table S4.** **Pairwise comparisons from weight gain in queens that received a pollen-starved diet with Tukey contrasts**. Queens were deprived of pollen (pollen-starved diets, hereafter “PS”) for some duration of time (either three, six, nine, or twelve days). Asterisks indicate statistical significance; significance codes: ‘***’ 0.001, ‘**’ 0.01, ‘*’ 0.05.

|  | **Tukey contrasts** | **Estimate** | **Standard Error** | **z-value** | **Pr(>\|z\|)** |
| --- | --- | --- | --- | --- | --- |
| **Treatment** | PS3d-control | 0.027 | 0.033 | 0.800 | 0.931 |
|  | PS6d-control | -0.036 | 0.033 | -1.088 | 0.813 |
|  | PS9d-control | -0.029 | 0.033 | -0.892 | 0.900 |
|  | PS12d-control | 0.017 | 0.034 | 0.514 | 0.986 |
|  | PS3d-PS12d | 0.009 | 0.034 | 0.278 | 0.999 |
|  | PS6d-PS12d | -0.053 | 0.033 | -1.619 | 0.485 |
|  | PS9d-PS12d | -0.047 | 0.033 | -1.403 | 0.626 |
|  | PS6d-PS3d | -0.063 | 0.033 | -1.891 | 0.322 |
|  | PS9d-PS3d | -0.056 | 0.033 | -1.696 | 0.436 |
|  | PS9d-PS6d | 0.007 | 0.032 | 0.204 | 1.000 |
| **Days** | 3-0 | 0.048 | 0.013 | 3.649 | 0.002 ** |
|  | 6-0 | 0.070 | 0.013 | 5.283 | < 0.001 *** |
|  | 9-0 | 0.076 | 0.013 | 5.729 | < 0.001 *** |
|  | 12-0 | 0.086 | 0.013 | 6.490 | < 0.001 *** |
|  | 6-3 | 0.022 | 0.013 | 1.634 | 0.475 |
|  | 9-3 | 0.028 | 0.013 | 2.080 | 0.229 |
|  | 12-3 | 0.038 | 0.013 | 2.841 | 0.036 * |
|  | 9-6 | 0.006 | 0.013 | 0.445 | 0.992 |
|  | 12-6 | 0.016 | 0.013 | 1.207 | 0.747 |
|  | 12-9 | 0.010 | 0.013 | 0.761 | 0.941 |

**Table S5.** **Pairwise comparisons from lipids concentration with Tukey contrasts**. Asterisks indicate statistical significance; significance codes: ‘*’ 0.05. Queens were deprived of either artificial nectar (nectar-starved diets, hereafter “NS”) or pollen (pollen-starved diets, hereafter “PS”) for some duration of time (either three, six, nine, or twelve days).

| **Tukey contrasts** | **Estimate** | **Standard Error** | **z-value** | **Pr(>\|z\|)** |
| --- | --- | --- | --- | --- |
| NS3d - CTL | 7.995 | 36.953 | 0.216 | 1.000 |
| NS6d - CTL | 19.883 | 38.914 | 0.511 | 0.999 |
| PS12d - CTL | 108.951 | 53.718 | 2.028 | 0.377 |
| PS3d - CTL | -27.130 | 27.670 | -0.980 | 0.954 |
| PS6d - CTL | 17.157 | 34.259 | 0.501 | 0.999 |
| PS9d - CTL | 111.996 | 49.009 | 2.285 | 0.236 |
| NS6d - NS3d | 11.889 | 42.701 | 0.278 | 1.000 |
| PS12d - NS3d | 100.956 | 55.570 | 1.817 | 0.516 |
| PS3d - NS3d | -35.125 | 32.965 | -1.066 | 0.932 |
| PS6d - NS3d | 9.162 | 38.770 | 0.236 | 1.000 |
| PS9d - NS3d | 104.001 | 51.720 | 2.011 | 0.388 |
| PS12d - NS6d | 89.068 | 57.165 | 1.558 | 0.691 |
| PS3d - NS6d | -47.013 | 35.082 | -1.34 | 0.821 |
| PS6d - NS6d | -2.727 | 40.168 | -0.068 | 1.000 |
| PS9d - NS6d | 92.112 | 52.860 | 1.743 | 0.567 |
| PS3d - PS12d | -136.081 | 50.716 | -2.683 | 0.094 |
| PS6d - PS12d | -91.795 | 54.808 | -1.675 | 0.614 |
| PS9d - PS12d | 3.044 | 64.136 | 0.047 | 1.000 |
| PS6d - PS3d | 44.287 | 29.772 | 1.488 | 0.736 |
| PS9d - PS3d | 139.126 | 45.763 | 3.040 | 0.035 * |
| PS9d - PS6d | 94.839 | 49.697 | 1.908 | 0.454 |

**Table S6.** **Pairwise comparisons from glycogen concentration with Tukey contrasts**. Queens were deprived of either artificial nectar (nectar-starved diets, hereafter “NS”) or pollen (pollen-starved diets, hereafter “PS”) for some duration of time (either three, six, nine, or twelve days).

| **Tukey contrasts** | **Estimate** | **Standard Error** | **z-value** | **Pr(>\|z\|)** |
| --- | --- | --- | --- | --- |
| NS3d - CTL | -0.224 | 0.246 | -0.909 | 1.000 |
| NS6d - CTL | -0.312 | 0.278 | -1.124 | 1.000 |
| PS12d - CTL | -0.458 | 0.239 | -1.914 | 1.000 |
| PS3d - CTL | -0.182 | 0.231 | -0.788 | 1.000 |
| PS6d - CTL | -0.656 | 0.239 | -2.747 | 0.126 |
| PS9d - CTL | -0.476 | 0.239 | -1.989 | 0.886 |
| NS6d - NS3d | -0.088 | 0.280 | -0.315 | 1.000 |
| PS12d - NS3d | -0.234 | 0.237 | -0.985 | 1.000 |
| PS3d - NS3d | 0.042 | 0.231 | 0.183 | 1.000 |
| PS6d - NS3d | -0.433 | 0.241 | -1.793 | 1.000 |
| PS9d - NS3d | -0.252 | 0.241 | -1.047 | 1.000 |
| PS12d - NS6d | -0.145 | 0.268 | -0.543 | 1.000 |
| PS3d - NS6d | 0.130 | 0.265 | 0.492 | 1.000 |
| PS6d - NS6d | -0.344 | 0.266 | -1.296 | 1.000 |
| PS9d - NS6d | -0.164 | 0.266 | -0.615 | 1.000 |
| PS3d - PS12d | 0.276 | 0.223 | 1.238 | 1.000 |
| PS6d - PS12d | -0.199 | 0.232 | -0.859 | 1.000 |
| PS9d - PS12d | -0.018 | 0.231 | -0.078 | 1.000 |
| PS6d - PS3d | -0.475 | 0.225 | -2.107 | 0.703 |
| PS9d - PS3d | -0.294 | 0.225 | -1.307 | 1.000 |
| PS9d - PS6d | 0.181 | 0.230 | 0.785 | 1.000 |

**Figure S1**


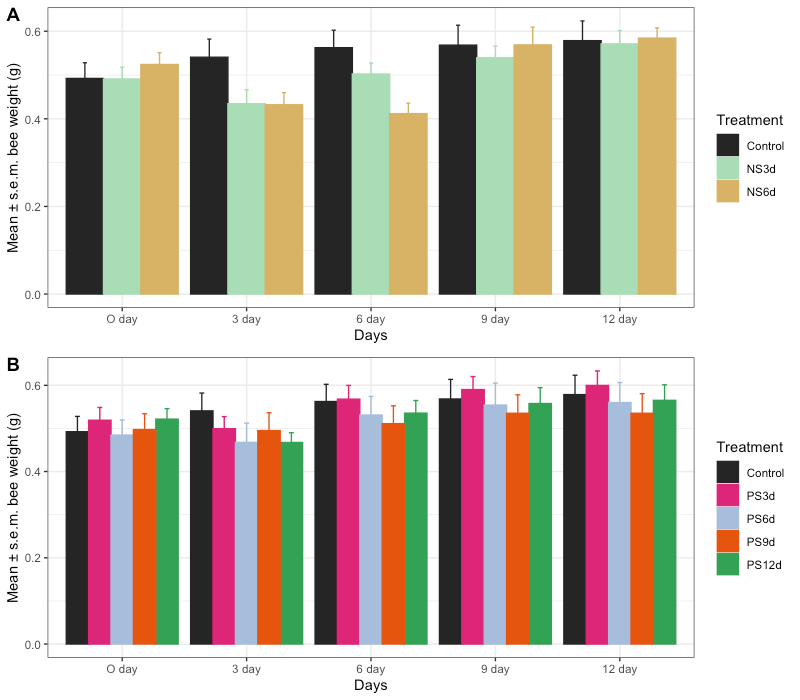


**Figure S1.** **Pairwise comparisons from weight change through time as a function of diet treatment.** Bee weight was recorded every three days and averaged within each treatment for each timepoint. A) Nectar starvation treatment groups, B) Pollen starvation groups. Data for the control diet group is shown (in black) for both A and B. Queens were deprived of either artificial nectar (nectar-starved diets, hereafter “NS”) or of pollen (pollen-starved diets, hereafter “PS”) for some duration of time (either three, six, nine, or twelve days). For nectar starvation, we found a significant weight difference between NS6d v control groups on day 6. No significant differences were detected between queen weights, when treatment groups were compared within a given day.
